# Estimation of transient increases in bleeding risk associated with physical activity in children with haemophilia

**DOI:** 10.1186/1471-2326-8-2

**Published:** 2008-06-26

**Authors:** Carolyn R Broderick, Robert D Herbert, Jane Latimer, Chris Barnes, Julie A Curtin, Paul Monagle

**Affiliations:** 1 School of Medical Sciences, University of New South Wales, Randwick NSW 2052, Australia; 2 Children's Hospital Institute of Sports Medicine, The Children's Hospital at Westmead, Westmead NSW 2145, Australia; 3 The George Institute for International Health, Camperdown NSW 2050, Australia; 4 Department of Haematology Royal Children's Hospital, Parkville VIC 3052, Australia; 5 Department of Haematology, The Children's Hospital at Westmead, Westmead NSW 2145, Australia; 6 Department of Pathology, University of Melbourne Parkville, VIC 3052, Australia

## Abstract

**Background:**

Although it is widely appreciated that vigorous physical activity can increase the risk of bleeding episodes in children with haemophilia, the magnitude of the increase in risk is not known. Accurate risk estimates could inform decisions made by children with haemophilia and their parents about participation in physical activity and aid the development of optimal prophylactic schedules. The aim of this study is to provide an accurate estimate of the risks of bleeding associated with vigorous physical activity in children with haemophilia.

**Methods/Design:**

The study will be a case-crossover study nested within a prospective cohort study. Children with moderate or severe haemophilia A or B, recruited from two paediatric haematology departments in Australia, will participate in the study. The child, or the child's parent or guardian, will report bleeding episodes experienced over a 12-month period. Following a bleeding episode, the participant will be interviewed by telephone about exposures to physical activity in the case period (8 hours before the bleed) and 2 control periods (an 8 hour period at the same time on the day preceding the bleed and an 8 hour period two days preceding the bleed). Conditional logistic regression will be used to estimate the risk of participating in vigorous physical activity from measures of exposure to physical activity in the case and control periods.

**Discussion:**

This case-control study will provide estimates of the risk of participation in vigorous physical activity in children with haemophilia.

## Background

Haemophilia affects 1 in 7,000 males in Australia.[1] Clinically, haemophilia is characterised by bleeds, most often into muscles or joints. Some children with hemophilia develop "target joints" when multiple bleeds into the same joint lead to destruction of the joint surfaces (haemophilic arthropathy).

Over the past decade there has been a move to treat children with moderate and severe haemophilia prophylactically. Typically a child may be given two to three intravenous injections of recombinant factor VIII or IX each week. Injections may be timed to precede vigorous physical activity because vigorous physical activity is thought to elevate the risk of bleeding episodes. Prophylactic clotting factor injections reduce the number of bleeding episodes, but many children with moderate and severe haemophilia still experience bleeding episodes several times a year.

Although it is widely believed that vigorous physical activity can increase the risk of bleeds, the magnitude of the increase in risk is not known. Accurate estimates of the risk of bleeding associated with physical activity would enable the tailoring of prophylactic schedules to individuals, taking into consideration fluctuations in risk.

In addition to guiding prophylactic schedules, good information about the risks of bleeds associated with vigorous physical activity is needed to inform the decisions made by children with haemophilia and their parents about participation in physical activities. Informed decisions must be supported by good estimates of risk.

This study will provide the first estimates of the risks of bleeding associated with vigorous physical activity in children with haemophilia. Risk estimates can form the basis of rational prophylactic schedules and can inform decisions about participation in physical activities.

### Aims

The aims of this study are, therefore, to:

(a) Quantify the transient increase in risk of bleeding associated with vigorous physical activity in children with haemophilia

(b) Determine whether habitual activity is associated with the transient risk of bleeding episodes following physical activity

(c) Determine the induction period for a bleed caused by vigorous physical activity

## Methods

### Design

The study will be a case-crossover study nested within a prospective cohort study. The case-crossover design enables quantification of the risk associated with transient exposures. It is more efficient than cohort designs because it samples only cases, and it is less exposed to selection bias than case-control designs because cases provide their own control data.

The target population is children with moderate or severe haemophilia A or B. To be eligible to participate, children must be aged between 4 and 18 years. Children with mild haemophilia (i.e. factor VIII or IX levels > 5%) or von Willebrand disorder will be excluded. Children will be recruited from two paediatric haematology departments in Sydney and Melbourne, Australia.

The aims and methods of the study will be explained to potential participants and their parents or guardians. Hereafter we refer to the child or parent or guardian as the participant. Participants will be given plain-English information sheets and will be asked to give written consent to participate. The study has been given ethical approval by the Human Research Ethics Committees at the University of Sydney, The Children's Hospital at Westmead and the Royal Children's Hospital, Melbourne.

### Procedures

Most interviews with participants will be conducted by telephone although where it is convenient to do so some interviews may be conducted in person. At the first interview a researcher will establish whether the interview is best conducted with the child or with the parent or guardian. The researcher will record participants' contact details and the child's characteristics, including current age, age at diagnosis, type of bleeding disorder, severity of bleeding disorder, estimated number of bleeds in the past 12 months, height and weight (BMI), prophylaxis schedule, habitual activity level, orthopaedic history (including history of known arthropathy and joint arthropathy score) and presence of neutralising antibodies or inhibitors (i.e. inhibitor titre of 0.5 Bethesda units or more [2]). Where necessary, and with permission from participants, these details will be obtained from the participant's medical record.

In addition, the participant will be asked to complete Kriska's modifiable activity questionnaire (MAQ).[3] This questionnaire will be administered directly to children over 12 years of age and to both child and parent in children less than 12 years of age. The questionnaire has been successfully used to measure habitual activity in children and adolescents with cystic fibrosis and has demonstrated test-retest reliability.[4] The questionnaire enables estimation of total hours of activity per week for the past year, average intensity (expressed as MET hours/week), and hours per week of vigorous activity (> 6 METs).

For the following year the participant will be monitored to determine whether a bleeding episode has occurred. Participants will be asked to notify the researchers of any bleeds that occur. Identification of bleeding episodes by self-report will be supplemented with two additional mechanisms: bleeding diaries and weekly SMS messages. All participants in the study will be given a bleeding diary. The diary will record the number and site of bleeding episodes. In addition each participant will be sent weekly SMS (text) messages. At a pre-specified time each week, a message will be sent to the participant to ascertain if a bleed has occurred in the past week. If no reply is received, follow up messages will be sent 24 and 48 hours later. If there is still no reply the participant will be contacted by phone. For the purposes of this study we have accepted the definition of a bleed as that widely used by other researchers, that is, "an episode of bleeding requiring treatment with clotting factor". [5]

If the participant reports a bleed in the preceding week the participant will be interviewed by telephone as soon as possible after the bleed. The timing of the interview and the bleed will be documented. The interviewer will construct an "activity clock" spanning the case and control periods. Information will be sought about the number, site, severity and timing of bleeds, treatment given, details of clotting factor injections administered over the previous two weeks, and details of any additional medication. This will provide information about:

• physical activity in the 8 hours immediately preceding the bleed *(exposure in the case period)*

• physical activity in the period 32-24 hours preceding the bleed *(exposure in the first control period)*

• physical activity in the period 56-48 hours preceding the bleed *(exposure in the second control period)*

Physical activity occurring in the case and control periods will be assigned to one of three categories reflecting the frequency and severity of collisions that children are expected to experience while participating in the activity. The categories will be based on those used by the American National Hemophilia Association in their popular brochure titled *Hemophilia Sport and Exercise*. Some additional activities (such as Australian Rules Football and netball) will be added to make the categories suitable for use with Australian populations. Category 1 activities are non-contact activities such as swimming and table tennis. (Walking will be excluded because it is ubiquitous and quantifying the risk of bleeding associated with walking is not of interest.) Category 2 activities involve limited contact such as soccer. Category 3 activities are those in which contact and collisions are inevitable (such as rugby). In addition questions will be asked about factors that are probably not risk factors. These data will be used in secondary analyses described below.

On many occasions the child will have experienced no bleeding episodes in the preceding week so an interview will not be required. Children who have already experienced a bleeding episode since entering the study will continue to be called weekly. The same procedures will be followed if there is a subsequent bleed, except if the subsequent bleeding episode occurs within a fortnight of a previous episode. In that case, the bleed will be counted for the purpose of documenting incidence of bleeds, but will be ignored when quantifying the risk of bleeds associated with physical activity.

On one occasion randomly selected during the year, participants will be contacted by telephone and asked the same questions about exposure to physical activity as they were asked following bleeds. These data will be used for the crossover-control analysis described below. Interviews conducted after bleeds may be hours or even days after the bleed, which means that interviews conducted after bleeds seek data about exposure periods the most recent of which was hours or even days prior to the interview. Consequently when conducting the interviews at randomly selected times (not after a bleed) participants will be asked about exposure periods, the most recent of which was hours or even days prior to the interview. At the same interview, participants will be asked to prospectively record all activity in the following week. This will provide prospectively collected data about the usual frequency of exposure that can be used in a usual frequency analysis.

Methods that could be used to blind participants and research assistants to the study aims were considered, but these methods were thought not to be effective or ethically acceptable. Blinding is desirable in case-control studies because it reduces the possibility of recall bias. However blinding may be less important in the case-crossover design. In the case-crossover design participants report exposure to physical activity in *both *the case and control periods. Recall bias is most problematic if there is *differential *mis-reporting in the case and control periods, which we believe is unlikely to occur. To minimise the possibility of reporting bias, participants will not be told that the primary aim is to quantify risks associated specifically with physical activity.

### Data analysis

Conditional logistic regression will be used to estimate the risk of participating in vigorous physical activity from measures of exposure to physical activity in the case and control periods. The basic regression model will be:

*log odds of bleed*_*i *_= *a*_*i *_+ *b.period *+ *c.severity*,

where *period *is a dummy-coded variable indicating if exposure to activity occurred in the exposure period or control period, and *severity *is a dummy-coded variable indicating if the subject had moderate or severe haemophilia. This analysis estimates an odds ratio (e^*b*^) which quantifies the risk of bleeding associated with vigorous physical activity. The analysis is conditional because data from the case period and the two control periods are matched, and because participants can experience multiple bleeds. In the primary analysis vigorous physical activity will be defined as Category 2 or 3 activities. Consequently this analysis will provide estimates of the risk of Category 2 or 3 activities.

In addition, the regression model will be extended to include terms for nonlinear effects of time since factor administration and their interactions with the *period *variable. This controls for time since factor administration, which we consider to be the most plausible potential confounder. [6] It will also provide useful information about the time course of risks associated with physical activity following factor administration.

In secondary analyses the risks associated with Category 1, Category 2 and Category 3 activities will be analysed separately. In addition, a usual frequency analysis will be conducted.[7] The usual frequency analysis will produce an alternative risk estimate by using negative binomial regression to contrast the incidence rate of exposures observed in the randomly selected week with the incidence rate of exposures in the case period. (If a bleeding episode occurred in the randomly selected week then only that part of the week more than 24 hours prior to the bleed will contribute to the estimate of the usual incidence rate of exposures).

If a participant wakes with a bleed, the time at which they would first have noticed symptoms of the bleed had they not been asleep is not known. Consequently an additional secondary analysis in which waking bleeds are excluded from the analysis will be performed.

We will test the hypothesis that the risk of bleeding associated with physical activity depends on the severity of disease by testing if an interaction term (*period *× *disease severity*) contributes significantly to the model.

Sensitivity analyses will be conducted by varying the length of case and control periods.

Data collected at times when participants did not bleed will be used to conduct a crossover-control analysis.[8] The crossover-control analysis compares exposures in two periods (a pseudo "case" period and a control period) that do not precede a bleed; this analysis should yield a risk estimate of approximately zero (odds ratio of ~1.0). This provides a test of the presence of reporting bias.

Data on the usual frequency of exposure to physical activity obtained from the randomly selected week will be combined with estimates of the transient relative risk associated with physical activity to estimate the cumulative risk associated with the usual frequency of physical activity.

To achieve the second aim of assessing whether habitual activity modifies the transient risk of bleeding episodes following physical activity, terms will be entered into the model for habitual physical activity (MAQ) measured at baseline, as well as the *period *× *habitual activity *interaction.

The primary analyses assume that the induction period for symptoms of a bleed caused by physical activity is less than 8 hours. Empirical evidence of the length of the induction period will be sought by examining histograms of the time elapsed between the onset of symptoms of each bleed and the last preceding occasion of physical activity. These histograms will be compared with histograms obtained from the control periods, and with the histograms obtained when participants were interviewed at one randomly selected occasion during the year, to determine the period over which physical activity appeared to contribute to an excess of bleeds (i.e. the induction period).

### Sample size calculations

The study is designed to be adequately powered for the primary analysis, which involves estimation of the risk of bleeding associated with physical activity. The approach to calculating sample size was as follows. First the sample size necessary for a paired case-control study was calculated using the procedures described by Dupont. [9] This showed that in a conventional paired case-control design with one event per participant we would need a sample of 210 cases (bleeds) to provide an 80% probability of detecting an odds ratio of 2.5 assuming (a) alpha is 0.05, (b) 20% of boys will be exposed to exercise in the control period, and (c) the correlation between exposures in the case and control period (phi coefficient) is 0.5. The relatively large risk (odds ratio of 2.5) is justified because the risk is only transient.

On the basis of bleeding rates observed at the Royal Children's Hospital haemophilia clinic it was anticipated that the participants will experience, on average, about 4 bleeding episodes per year. The sample size estimate was therefore divided by 4 to obtain a crude estimate of the required number of participants. Then, to allow for the lack of independence amongst repeated bleeds, the sample size was inflated by a factor that was a function of the estimated average number of bleeds per participant (four) and an estimated maximum intra-class (intra-participant) correlation (0.2). [10] This yielded a sample size of 336 bleeds. It is expected that this sample size will be achieved with 84 participants, but the study will be terminated with the 336th bleed, not the 84th participant.

## Discussion

This project will provide the first research-based estimates of the risk of bleeding episodes associated with vigorous physical activity in children with haemophilia. These risk estimates can be used to inform decisions about participation in vigorous physical activities. Estimates of the risks of bleeds associated with particular categories of physical activity can be used to appropriately weigh risks and benefits of participation in vigorous physical activities. The design of optimal prophylactic schedules requires information about the magnitude of the fluctuations in risk associated with physical activity. Good risk data can therefore be used to optimise prophylactic strategies.

## Abbreviations

SMS: short messaging service; Haemophilia A: Factor VIII deficiency; Haemophilia B: Factor IX deficiency; BMI: Body Mass Index; MAQ: Modifiable Activity Questionnaire; MET: Metabolic Equivalents.

## Competing interests

The authors declare that they have no competing interests.

## Authors' contributions

CRB and RDH conceived the study and were involved in study design and preparation of the manuscript. JL was involved in study design and preparation of the manuscript. CB, PM and JAC were involved in the study design and advice regarding relevant clinical details of children with haemophilia. All authors read and approved the final manuscript.

## Pre-publication history

The pre-publication history for this paper can be accessed here:


